# Food insecurity, home ownership and income-related equity in dental care use and access: the case of Canada

**DOI:** 10.1186/s12889-022-12760-6

**Published:** 2022-03-14

**Authors:** Margherita Giannoni, Michel Grignon

**Affiliations:** 1 Department of Economics, University of Perugia, Perugia, Italy; 2 Department of Management, Scuola Superiore S. Anna, Pisa, Italy; 3 Department of Economics, McMaster University, Hamilton, ON Canada; 4 Department of Health, Aging and Society, McMaster University, Hamilton, ON Canada

**Keywords:** Equity health care utilization, Dental care, Food insecurity, I10, I11, I14, I19

## Abstract

**Background:**

It has been documented that income is a strong determinant of dental care use in Canada, mostly due to the lack of public coverage for dental care. We assess the contributions of food insecurity and home ownership to income-related equity in dental care use and access. We add to the literature by adding these two variables among other socio-economic determinants of equity in dental care use and access to dental care. Evidence on equity in access to and use of dental care in Canada can inform policymaking.

**Methods:**

We estimate income-related horizontal inequity indexes for the probability of 1) receiving at least one dental visit in the last 12 months; and 2) lack of dental visits during the 3 years before the interview. We conduct the analyses using data from the 2013–2014 Canadian Community Health Survey (CCHS) at the national and regional level.

**Results:**

There is pro-rich inequity in the probability of visiting a dentist or an orthodontist and in access to dental care in Ontario. Inequities vary across jurisdictions. Housing tenure and food insecurity contribute importantly to both use of and access to dental care, adding information not captured by standard socio-economic determinants.

**Conclusions:**

Redistributing income may not be enough to reduce inequities. Careful monitoring of equity in dental care is needed together with interventions targeting fragile groups not only in terms of income but also in improving house and food security.

**Supplementary Information:**

The online version contains supplementary material available at 10.1186/s12889-022-12760-6.

## Background

There is some evidence of socio-economic inequalities in oral health [1, 2]. Pro-rich socio-economic inequalities and income-related inequities in the utilization of some health care services in Canada, particularly dental care, have been documented [3–10]. Also, during and after the global financial crisis of 2008–9, food insecurity and housing problems have increased in Canada as well as in the US and Europe [11, 12]. There are good reasons to believe that food insecurity and home ownership may affect the extent of social inequalities and income-related inequity in dental care use in Canada. In this study, we propose to enrich the description of socio-economic status (SES) in assessing equity in access and utilization of dental care by adding these two measures of household economic and financial insecurity as non-need independent variables beside income. Annual income provides much information on what commodities a household can afford and, as a result, whether the household can afford dental care services without jeopardizing its other consumption. We posit that it does not, however, capture all relevant information, and we propose to enrich the description of SES by adding these two household characteristics. Food insecurity is a measure of how likely it is that a household cannot afford the required quantity and quality of food [11]. A general definition of household food insecurity is given by Anderson [13] and refers to having a limited or uncertain availability of nutritionally adequate and safe food, or to having to acquire foods in socially unacceptable ways. This is a measure of purchasing power, and we posit that purchasing power may vary across households with similar income levels, especially since the big recession of 2008. The second characteristic is home ownership: here, we hypothesize that two households with the same income may have different purchasing power and situations relative to uncertainty depending on whether they own their house, especially in Canadian urban centres where housing prices have increased dramatically [14, 15]. As a result, home ownership status may affect the affordability of dental care services, conditional on income, and we want to test this is the case. There is evidence for both Canada and the US that both food insecurity [16–25] and housing insecurity [26–29] have a negative impact on individual physical and mental health. Duncan and Bonner [30] for Canada find a detrimental effect of food insecurity on oral health. Access to healthy food is considered a factor that affects oral health [31, 32]. There is conflicting evidence on the link between food insecurity and health care use. Tarasuk et al. find an effect for Canada for ambulatory care [33] and for mental health care [25]. Kushel et al. [26] find an effect for the US and both ambulatory and emergency care. However, Bhargava and Lee [34] find no effect among the 50+ in Georgia, US. On dental care in particular, Allin et al. [5] find that, in Europe and the US, inequalities in dental care use are more pronounced across wealth than income quintiles. Muirhead et al. [35] show that food insecurity has a powerful impact on dental care use among Ontario working poor: 40% of those who are food insecure report seeing a dentist only when in pain, versus 18% of those who are food secure; 52% of those who are food insecure reported unmet dental care need, versus 24% of those who are food secure. Previous work on unmet needs for dental care in Canada found evidence that individuals with low income and without dental insurance coverage had the highest probability of reporting financial barriers to dental care and that reported unmet needs due to costs were associated with lower dental visit frequency and poorer oral health outcomes after controlling for the effects of income and insurance coverage [36]. However, the impact of both the lack of house ownership and food insecurity on income-related inequities in dental care use and unmet access to dental care has never been studied. We will add to the literature on income-related inequities by introducing these two variables among other socio-economic (non-need) determinants and by estimating their contribution on horizontal equity in dental care use. We will also add a rich set of controls describing chronic conditions, testing their role on dental care utilization. It has been shown for other countries like Italy that equity in the use of and unmet needs for health care services may vary not only by region [37] but also by type of chronic disease [38]. There is evidence for Canada that the use of health care services varies with the type of chronic conditions [4] and that being affected by diabetes tends to be associated with lower access to dental care [10].

However, evidence is needed on the role of food insecurity and home ownership in income-related inequities in access to dental care in Canada.

## Data

The data used were from the 2013–2014 Canadian Community Health Survey (CCHS) annual component, conducted by Statistics Canada [39]. This is a large survey representative of the Canadian population and at the provincial level.

CCHS includes both a mandatory core set of questions that are completed by respondents in all provinces and a set of optional components completed at the discretion of individual provinces. We based our analysis on the full national sample with non-missing values on all the variables used and the adult population aged more than 17 years old. We firstly estimated models for the probability of using dental care by using CCHS data for all Canadian main regions. For our purposes, respondents living in Ontario (approximately one-third of the population) were asked questions on unmet need for dental care, which we used to study access to dental care for that province only and we estimated models for the probability of reporting unmet need for dental care with the richer set of independent variables on dental care that was only available for Ontario (dental care inclusion modules 1 and 2 of the CCHS). The details of the selection process of the datasets used are reported in additional online resources (Additional file 1).

### Dependent variables

Our two dependent variables measured dental health care use and lack of access to dental care. The measure for dental care utilization was based on a standard question regarding whether individuals consulted a dentist, dental hygienist or orthodontist during the 12 months preceding the interview (consulting meaning either seen or talked to).^1^ To study access, we used a question posed to respondents living in Ontario (and Northwest Territories) only, relative to not having visited a dentist over the past 3 years. Respondents who reported no visit in the past 3 years were classified as having unmet access to dental care, under the assumption that any individual should visit a dentist at least once every 3 years. We also used a more subjective variable in a sensitivity analysis: those who reported no visits in 3 years were asked the reason for this, and we created a variable of unmet access for financial reasons by giving a value of 1 to respondents stating the reason was due to costs. Last, we used a question posed to respondents in Ontario only to run a sensitivity analysis on our analysis of dental care use (first analysis): “*It was reported earlier that you have ‘seen’ or ‘talked to’ a dentist in the past 12 months. Did you actually visit one?”,* the variable on utilization in the past 12 months being restricted to those who had visited one*.*

### Independent variables

#### Need variables

The need variables included age, gender, poor self-assessed health, poor self-assessed mental health, and low perceived life satisfaction. Following previous studies [38], we tested the inclusion of additional chronic health indicators, including a dummy set for the main chronic diseases reported by respondents (rheumatic diseases, cardiovascular, respiratory, diabetes, cancer, digestive diseases, back problems and scoliosis, anxiety, and other chronic diseases).^2^ After this, we kept the only chronic condition variable that had a significant impact in all our models, having been diagnosed with diabetes. Unfortunately, information on oral health (a potential important determinant of the need for dental health care) was available only for respondents living in Ontario. We ran a sensitivity analysis on that sub-population, including information on oral health, and concluded that including it did not change the statistical significance or values of the other estimated effects. For analyses conducted on respondents from Ontario, we used the following variable for oral health: we constructed an index for oral health status by Cronbach’s alpha, pooling a set of 14 questions on oral health conditions.^3^ We then used a variable which takes the value of 1 if the individual belongs to the lowest tertile of the distribution of this index, reporting a poor dental health status, and zero otherwise.

#### Non-need variables

We divided income by the square root of household size and then took the logarithm of this equalized income. Food insecurity (FI) is measured by Statistics Canada with an indicator on Household Food Insecurity Status, based on a set of 18 questions, and describes the food security situation of the household in the previous 12 months. It captures three kinds of situations: 1–Food secure: no sign of difficulty with income-related food access; 2–Moderately food insecure: sign of compromised quality and/or quantity of food consumed; 3–Severely food insecure: sign of reduced food intake and disrupted eating patterns. This variable is adopted from the Health Canada model of food security status. FI is measured in the US and in Canada with a standard questionnaire. We used data from the Group Food Security Module of the CCHS survey. This is based on the definition of FI as “The uncertainty and insufficiency of food availability and access that are limited by resource constraints, and the worry or anxiety and hunger that may result from it” (Wunderlich and Norwood, p.49) [40]. Those households classified as food secure did not report issues. Households classified as moderately food insecure had problems in the quality and/or quantity of food consumed among adults and/or children, whereas severely food insecure showed more extensive compromises, including reduced food intake among adults and/or children because of a lack of money for food (see Statistics Canada [39]; Tarasuk [20]).^4^ We therefore used two dummy variables if the individuals lived respectively in a severely or moderately food insecure family, and zero otherwise. Housing status in the survey was captured by a question relative to house tenure status, where individuals were asked if they were living in a rented home or had home ownership. We used the dummy for home rented as a proxy for house insecurity, as opposed to house security with home ownership. There was no possibility to distinguish between those owning their home with or without debt. In our estimates we controlled for a set of further non-need variables such as marital status, immigrant status, race/ethnicity (aboriginal, non-white), smoking status (daily smoking), obesity, regular alcohol consumption, and living in rural areas. The provinces in which individuals live were collapsed into six regions, and a dummy variable for each one was created: (1) Atlantic, which includes Nova Scotia, New Brunswick, Prince Edward Island and Newfoundland-Labrador; (2) Quebec; (3) Prairies, which includes Alberta, Saskatchewan and Manitoba; (4) Territories, which includes Yukon, Northwest Territories and Nunavut; (5) British Columbia; and (6) Ontario.

We added one variable related to dental hygiene lifestyle. In the special module available only for Ontario, individuals were also asked their frequency of brushing teeth (“*How often do you brush your teeth?*”). We used a dummy variable *that t*akes the value of 1 if this was at least twice a day, and zero otherwise. Last, we used another dummy, taking the value of 1 if individuals were covered by dental insurance, and zero otherwise. Our reference individual for the models on dental visits utilization was a white local citizen, married (or common-law) middle income and middle-aged (46–55 years) woman, living in Ontario, not actively working as employee or self-employed i.e. being either unemployed, housework, student, receiving social support or in other non-active conditions, with secondary education level, with at least a fair health/mental health/life satisfaction status, not affected by diabetes, not obese, neither currently smoking nor practicing heavy drinking, not physically active, not food insecure, not having to pay rent for a home, living in an urban area. For the models on unmet access to dental visits, the reference individual had the same characteristics as above and, in addition, had at least a fair or good oral health, no dental insurance, and did not brush her/his teeth at least twice a day. Descriptive statistics for the main dependent and independent variables used are shown in Table 1.

**Table 1:** Variables and descriptive statistics

**Variables**		**Sample size**^**a**^	**Weighted %**^**b**^
**Dependent variables:**			
*Dental or orthodontal care in the last 12 months*	YES	33,712	66%
	NO	17,367	34%
*Unmet access to dental visits (last time visited dentist replied 3 years or more) (Ontario)*^*c*^	YES	2318	14%
	NO	14,236	86%
**A) Need variables**			
*Self- assessed health status*	POOR OR VERY POOR	5619	11%
	FAIR, GOOD OR VERY GOOD	45,460	89%
*Self- assessed mental health status*	POOR OR VERY POOR	3070	6%
	FAIR, GOOD OR VERY GOOD	48,102	94%
*Self-assessed dental health status (Ontario)*^*c*^	POOR	329	2%
	FAIR, GOOD OR VERY GOOD	16,225	98%
*Age*	*18–25*	7151	14%
	*26–35*	8683	17%
	*36–45*	9194	18%
	*46–55*	9194	18%
	*56–65*	8683	17%
	*65+*	8173	16%
*Sex*	MALE	25,029	49%
	NON MALE	26,050	51%
*Chronic conditions:*			
*Diabetes*	YES	3576	7%
	NO	47,503	93%
**B) Non-need variables**			
*Lifestyles:*			
*Obese*	YES	10,216	20%
	NO	40,863	80%
*Daily smoker*	YES	7151	14%
	NO	43,928	86%
*Regular drinker*	YES	32,691	64%
	NO	18,388	36%
*Brush teeth at least 2 times a day (Ontario)*^*c*^	YES	12,747	77%
	NO	3807	23%
*Other socioeconomic:*			
*Migrant status*	*IMMIGRANT*	13,281	26%
	*NOT IMMIGRANT*	37,798	74%
*Cultural racial background:*	*NON-WHITE*	10,216	20%
	*WHITE*	40,863	80%
*Aboriginal identity*	YES	1532	3%
	NO	49,547	97%
*Level of education:*	LESS THAN SECONDARY	6129	12%
	SECONDARY	10,216	20%
	SOME POST SECONDARY	3065	6%
	POST-SECONDARY CERTIFIED	31,669	62%
*Occupational status:*	EMPLOYED	28,604	56%
	SELF-EMPLOYED	5619	11%
	OTHER (RETIRED, UNEMPLOYED, ETC.)	16,856	33%
*Housing tenure status: rented house*	YES	13,791	27%
	NO	37,288	73%
*Marital status:*	WIDOW/SEPARATED/ DIVORCED	6640	13%
	SINGLE	12,259	24%
	MARRIED	26,050	51%
	COMMON LAW	6129	12%
*Household food insecurity:*	NO FOOD INSECURITY	48,014	94%
	MODERATE	2043	4%
	HIGH	1022	2%
*Dental insurance coverage (Ontario)*^*c*^	YES	7780	47%
	NO	8774	53%
*Geographic factors:*	RURAL	9194	18%
	URBAN	41,885	82%
	ATLANTIC	3517	7%
	QUEBEC	11,956	23%
	BRITISH_COL	6719	13%
	PRAIRIES	9186	18%
	TERRITORIES	151	0%
	ONTARIO	19,550	38%
***C) Other socioeconomic continuous variables***	***Description***	***Mean***	***St. Dev***
*Income*	*HOUSEHOLD TOTAL INCOME (CAD)*	89,139	300,081
*Household size*	*HOUSEHOLD SIZE*	2.82	1.43
*Equalized income*	*=INCOME/SQRT (HOUSEHOLD SIZE)*	54,857	174,678
*Natural log of equalized income*	*=LN (EQUALIZED INCOME)*	10.6	0.95

Data source: CCHS 2013–2014

^a^Weighted statistics. Total sample size with non-missing observations was 51,079 (see Additional file 1)

^b^Weighted proportions

^c^Weighted statistics. Ontario special Dental module sample size with non-missing observations was 16,554

## Methods

According to the principle of horizontal equity in health care, access should depend only on need, while socio-economic factors unrelated to need should not influence utilization [41]. We tested for income-related equity in health care use, applying the indirect standardization by regression method [41–45]. The horizontal inequity index (HI) is defined as the difference between income-related inequality in the observed health care use (CM) and income-related inequality in “need-expected” use (CN). The need-expected use is obtained by setting at their sample mean all non-need variables. The concentration index CM is positive (negative) whenever better-off individuals (worse-off) use dental care more than worse-off individuals (better-off). If the need distribution is favouring those who are worse off (better off), then we observe that the value of CN is negative (positive). There is no inequity in access to care when CM equals CN or the difference is not statistically significant. A positive (negative) value of the inequity implies inequity favouring those who are better off (worse off). Conversely, when the dependent variable measures unmet access to dental care, a negative (positive) value of HI implies inequity favouring those who are better off (worse off). An intuitive interpretation of the HI results can be obtained by multiplying HI by 75; for example, a HI value of 0.2 implies that equity can be achieved by redistributing 15% (0.2 × 75) of care from the rich to the poor [41]. For more details on the methodology see the additional resource (Additional file 2). In order to estimate CN, we estimated two set of logit models for: 1–the probability of having consulted a dentist or an orthodontist in the last 12 months in Canada; 2–the probability of having visited a dentist or an orthodontist in the last 12 months and of reporting lack of access to dental visits in the last 3 years in Ontario. The model for the lack of access to dental visits in Ontario was also estimated for the subset of individuals that indicated the main reason for not having visited a dentist in the last 3 years to be dental care costs. In all models we distinguished independent variables between need and non-need variables (Table 1). Non-need variables were set to their sample mean for obtaining CN [41, 45]. We estimated the income-related HI index for 1) the probability of using dental care services (all Canada and provinces) and 2) for the probability of unmet access to dental visits in the last 3 years (Ontario). All estimates were derived from weighted models using the population weights provided by Statistics Canada with the survey data. The estimated standard errors were based on the bootstrap method using weights provided by Statistics Canada.

## Results

The proportion of Canadians visiting a dentist in 2013–2014 was approximately 67%; the proportion of those who reported they did not access a dental visit in the last 3 years—our measure for unmet access to dental care^1^—in Ontario and Northwest Territories was 16% (Table 1), and 14% in Ontario alone.^5^ Of all individuals in Canada, 6% were living in food insecurity (4% moderate and 2% severe) (Table 1). Those living in a rented house were 27% of the total population, but this proportion more than doubles among the moderately food insecure (63%) and is almost three times higher among the severely food insecure (75%). Those who reported dental visits in the past 12 months were slightly less likely to be moderately (3.9%) or severely (1.6%) food insecure than the general population, and only 19% were tenants. These proportions were much higher among individuals reporting not having seen a dentist in the last 3 years in Ontario, of which approximately 17% were moderately (11%) or severely (6%) food insecure and 44% were tenants. Among those who reported unmet access, 30% were tenants and had food insecurity (either moderate or severe), i.e., five times the proportion of those who did not report problems in access (6%).

Overall, individuals who were better off were more likely to use dental care than the poor and less likely to report unmet access to dental visits during the 3 years preceding the interview. Tables 2 and 3 show the estimated odds ratios for all the logistic models. As expected, the probability of visiting a dentist or an orthodontist in Canada and of reporting problems in access to dental care is overall significantly associated with health care needs. With respect to the reference individual, being male, younger or elderly, being obese, and having diabetes are all factors associated with lower odds of use and higher odds of unmet access to dental care. Higher income is associated with higher odds of using dental care and conversely of unmet access to dental care, as high education and being employed. People with a high level of education and living in British Columbia were also more likely to access dental care. Ethnicity, marital status, and lifestyle all have associations with dental care access: being non-white or aboriginal, being a widow, being obese, smoking, and regular alcohol use are all associated with lower odds of using dental care. Engaging in regular physical exercise is associated with higher odds of visiting a dentist.

**Table 2:** Logit estimates for the probability of using dental visits by geographic areas (Odds Ratios)^a^

**Variables**	**Ontario**					**Atlantic**					**Quebec**				
	*Odds Ratio*	*z*	*P > |z|*	*[95% Conf. Interval]*		*Odds Ratio*	*z*	*P > |z|*	*[95% Conf. Interval]*		*Odds Ratio*	*z*	*P > |z|*	*[95% Conf. Interval]*	
*Health and lifestyles:*															
Poor or very poor self-assessed health status (ref good or fair)	0.74	−2.6	0.009	0.598	0.928	0.90	−1	0.341	0.718	1.121	0.86	−1.2	0.221	0.667	1.098
Affected by diabetes (ref. no diabetes)	0.83	−1.6	0.107	0.657	1.042	0.86	−1.3	0.191	0.684	1.079	0.75	−2.3	0.023	0.580	0.960
Obese (ref. Not obese)	0.92	−1	0.325	0.784	1.084	0.81	−2.2	0.030	0.674	0.980	0.93	−0.7	0.477	0.751	1.143
Current smoker (ref. Non-smoker or past smoker)	0.56	−5.7	0.000	0.461	0.684	0.64	−3.7	0.000	0.503	0.811	0.67	−3.9	0.000	0.544	0.818
Regular drinker (ref. Non-drinker or, occasional drinker)	1.48	4.68	0.000	1.257	1.748	1.59	5.3	0.000	1.340	1.888	1.66	5.27	0.000	1.374	2.002
*Immigration and Ethnicity:*															
Immigrant (ref. Local)	0.78	−2.4	0.018	0.629	0.956	0.93	−0.3	0.756	0.586	1.475	0.89	−0.7	0.478	0.643	1.229
Non-white (ref. White)	0.87	−1.1	0.291	0.678	1.124	0.77	−0.9	0.390	0.421	1.402	0.87	−0.7	0.459	0.594	1.266
Aboriginal (ref. Non aboriginal)	0.86	−0.9	0.368	0.613	1.199	0.92	−0.5	0.597	0.682	1.246	0.86	−0.7	0.475	0.562	1.309
Education level low education secondary (ref.)	0.49	−6	0.000	0.390	0.619	0.50	−5.5	0.000	0.388	0.637	0.54	−5.2	0.000	0.433	0.685
Education level high	1.21	2.02	0.044	1.005	1.455	1.16	1.59	0.112	0.965	1.402	1.19	1.8	0.072	0.985	1.428
*Material resources:*															
Living in a house rented (ref. home owner)	0.57	−6.2	0.000	0.480	0.683	0.79	−2	0.044	0.629	0.994	0.73	−2.9	0.004	0.588	0.903
Food insecurity:															
No Food insecurity (ref.) Moderately food insecure	0.59	−3.3	0.001	0.435	0.810	0.60	−2.5	0.013	0.405	0.898	0.78	−1	0.297	0.491	1.243
Highly Food insecure	0.73	−1.4	0.150	0.480	1.119	0.89	−0.3	0.733	0.454	1.742	0.40	−2.8	0.005	0.210	0.758
Equivalized personal Income (log)	1.24	2.51	0.012	1.049	1.476	1.62	5.84	0.000	1.381	1.912	1.57	4.62	0.000	1.296	1.900
*Geographical factors:*															
Living in a rural area (ref. urban area)	0.77	−3.4	0.001	0.663	0.898	0.89	−1.4	0.159	0.760	1.046	0.71	−3.8	0.000	0.600	0.851
Atlantic															
Quebec															
British_Col															
Prairies															
Territories															
*Statistics:*															
N	16,546					6619					9650				
k	26					26					26				
chi2	624					451.223					524				
p	0					0					0				
CN	0.001	0.81	0.416	−0.000	0.002	−0.001	0.00	−3.070	0.002	−0.01	0.012	7.2	0.000	0.006	0.011
CM	0.113	22.7	0.000	0.104	0.123	0.141	0.01	17.860	0.000	0.125	0.141	17.9	0.000	0.125	0.156
HI	0.113	22.8	0.000	0.103	0.123	0.144	0.01	18.440	0.000	0.129	0.129	16.8	0.000	0.113	0.148
**Variables**	**Prairies**					**Territories**					**B. Columbia**				
	*Odds Ratio*	*z*	*P > |z|*	*[95% Conf. Interval]*		*Odds Ratio*	*z*	*P > |z|*	*[95% Conf. Interval]*		*Odds Ratio*	*z*	*P > |z|*	*[95% Conf. Interval]*	
*Health and lifestyles:*															
Poor or very poor self -assessed health status (ref good or fair)	0.62	−4	0.000	0.487	0.780	0.55	−2.5	0.014	0.344	0.887	1.04	0.22	0.823	0.743	1.452
Affected by diabetes (ref. no diabetes)	1.14	0.91	0.365	0.861	1.502	0.99	−0	0.989	0.482	2.051	0.92	−0.4	0.682	0.631	1.352
Obese (ref. Not obese)	0.94	−0.6	0.527	0.780	1.136	1.03	0.22	0.826	0.767	1.394	0.69	−2.7	0.008	0.532	0.907
Current smoker (ref. Non-smoker or past smoker)	0.61	−4.6	0.000	0.492	0.753	0.87	−0.7	0.492	0.593	1.286	0.61	−3.2	0.002	0.448	0.828
Regular drinker (ref. Non-drinker or, occasional drinker)	1.25	2.49	0.013	1.049	1.495	1.27	1.35	0.179	0.895	1.813	1.26	2.1	0.036	1.015	1.554
*Immigration and Ethnicity:*															
Immigrant (ref. Local)	0.95	−0.4	0.665	0.741	1.211	1.24	0.64	0.522	0.637	2.427	0.84	−1.5	0.136	0.662	1.058
Non-white (ref. White)	0.74	−1.8	0.070	0.541	1.025	0.56	−1.6	0.102	0.274	1.124	0.95	−0.3	0.744	0.722	1.262
Aboriginal (ref. Non aboriginal)	0.64	−2.5	0.014	0.450	0.915	0.94	−0.3	0.770	0.613	1.438	1.32	1.04	0.296	0.786	2.202
Education level low education secondary (ref.)	0.71	−2.6	0.010	0.547	0.922	1.48	1.27	0.203	0.810	2.689	0.47	−4.8	0.000	0.345	0.639
Education level high	1.18	1.79	0.074	0.984	1.414	1.49	1.92	0.054	0.992	2.248	1.26	1.88	0.060	0.990	1.593
*Material resources:*															
Living in a house rented (ref. home owner)	0.77	−2.6	0.010	0.626	0.938	0.69	−1.9	0.057	0.469	1.012	0.65	−3.3	0.001	0.506	0.845
Food insecurity:															
No Food insecurity (ref.) Moderately food insecure	0.74	−1.4	0.168	0.479	1.137	0.68	−1.3	0.208	0.374	1.239	n.a				
Highly Food insecure	0.78	−0.6	0.566	0.329	1.839	1.22	0.41	0.680	0.477	3.106	n.a				
Equivalized personal Income (log)	1.34	4.07	0.000	1.162	1.535	1.38	2.83	0.005	1.103	1.714	1.38	3.71	0.000	1.163	1.631
*Geographical factors:*															
Living in a rural area (ref. urban area)	0.93	−0.7	0.499	0.757	1.145	1.09	0.38	0.703	0.712	1.655	0.70	−2.7	0.007	0.546	0.908
*Statistics:*															
N	10,758					1298					6269				
k	26					26					26				
chi2	267					67.056					229.3				
p	0					0					0				
CN	0.012	6.48	0.000	0.005	0.009	0.000	−0.1	0.960	−0	0.003	0.002	2.21	0.027	0.002	0.004
CM	0.105	12.9	0.000	0.089	0.121	0.123	6.14	0.000	0.084	0.163	0.095	11.4	0.000	0.079	0.112
HI	0.098	12	0.000	0.076	0.108	0.123	5.39	0.000	0.071	0.152	0.093	11.1	0.000	0.077	0.11
**Variables**	**Canada**														
	*Odds Ratio*	*z*	*P > |z|*	*[95% Conf. Interval]*											
*Health and lifestyles:*															
Poor or very poor self assessed health status (ref good or fair)	0.79	−3.8	0.000	0.703	0.892										
Affected by diabetes (ref. no diabetes)	0.87	−2.2	0.026	0.765	0.983										
Obese (ref. Not obese)	0.89	−2.6	0.010	0.810	0.972										
Current smoker (ref. Non smoker or past smoker)	0.62	−9	0.000	0.560	0.689										
Regular drinker (ref. Non driker or, occasional drinker)	1.43	7.88	0.000	1.311	1.568										
*Immigration and Ethnicity:*															
Immigrant (ref. Local)	0.88	−2	0.042	0.773	0.995										
Nonwhite (ref. White)	0.84	−2.2	0.031	0.720	0.984										
Aboriginal (ref. Non aboriginal)	0.84	−1.9	0.053	0.706	1.003										
Education level low education secondary (ref.)	0.54	−9.7	0.000	0.482	0.615										
Education level high	1.23	4.37	0.000	1.121	1.351										
*Material resources:*															
Living in a house rented (ref. Home owner)	0.66	−8	0.000	0.599	0.732										
Food insecurity:															
No Food insecurity (ref.) Moderately food insecure	0.66	−3.9	0.000	0.540	0.816										
Highly Food insecure	0.64	−3.1	0.002	0.475	0.850										
Equivalized personal Income (log)	1.35	5.7	0.000	1.219	1.501										
*Geographical factors:*															
Living in a rural area (ref. urban area)	0.80	−5.1	0.000	0.729	0.868										
Region:															
Ontario (ref.)															
Atlantic	0.59	−9.6	0.000	0.53	0.657										
Quebec	0.62	−8.6	0.000	0.56	0.695										
British_Col	0.81	−3.4	0.001	0.715	0.915										
Prairies	0.54	−12	0.000	0.486	0.599										
Territories	0.83	−1.6	0.121	0.65	1.051										
*Statistics:*															
N	51,079														
k	31														
chi2	1903.7														
p	0														
CN	0.004	10.7	0.000	0.004	0.005										
CM	0.116	36.1	0.000	0.109	0.122										
HI	0.111	35	0.000	0.105	0.117										

Bootstrap Normal-based standard errors. Data source: CCHS 2013–2014

^a^Estimates obtained after controlling for age, sex, marital status, urbanization level, occupational status

**Table 3:** Logit models for dental care use and for unmet access to dental care use – Ontario^a^

**Variables**	**Has seen a dentist or orthodontist in the last 12 months**						**No dental visits during the last 3 years**						**No dental visits during the last 3 years- reason:Costs**					
	*Odds*	*Bootstrap*		*P > |z|*	*Normal-based*		*Odds*	*Bootstrap*		P > |z|	*Normal-based*		*Odds*	*Bootstrap*		*P > |z|*	*Normal-based*	
	*Ratio*	*Std. Err.*	*z*		*[95% Conf. Interval]*		*Ratio*	*Std. Err.*	*z*		*[95% Conf. Interval]*		*Ratio*	*Std. Err.*	*z*		*[95% Conf. Interval]*	
*Health and lifestyles:*																		
Poor or very poor self-assessed dental health status (ref good or fair	1.07	0.0885	0.81	0.417	0.909	1.2579	0.82	0.0927	−1.72	0.086	0.6611	1.0277	0.60	0.1209	−2.54	0.011	0.4036	0.8898
Brushe teeth at least twice a day	1.42	0.1368	3.66	0.000	1.178	1.7170	0.59	0.0751	−4.16	0.000	0.4572	0.7546	0.67	0.1279	−2.08	0.038	0.4652	0.9781
Affected by diabetes (ref. no diabetes)	0.80	0.1077	−1.66	0.097	0.614	1.0411	1.32	0.2422	1.52	0.127	0.9234	1.8933	2.06	0.5377	2.77	0.006	1.2361	3.4368
Obese (ref. Not obese)	0.91	0.0867	−1.02	0.306	0.7520	1.0936	0.87	0.1146	−1.08	0.282	0.6697	1.1240	0.95	0.1879	−0.27	0.789	0.6431	1.3983
Current smoker (ref. Non-smoker or past smoker)	0.59	0.0645	−4.81	0.000	0.479	0.7337	1.65	0.2005	4.09	0.000	1.2962	2.0897	1.54	0.2762	2.41	0.016	1.0839	2.1890
Regular drinker (ref. Non driker or, occasional drinker)	1.43	0.1327	3.87	0.000	1.193	1.7163	0.71	0.0799	−3.04	0.002	0.5695	0.8852	0.89	0.1598	− 0.65	0.513	0.6250	1.2644
*Immigration and Ethnicity:*																		
Immigrant (ref. Local)	0.85	0.0976	−1.44	0.150	0.676	1.0617	1.00	0.1512	0.03	0.980	0.7472	1.3486	1.29	0.2929	1.13	0.259	0.8280	2.0144
Nonwhite (ref. White)	0.85	0.1190	−1.18	0.238	0.643	1.1158	1.18	0.2130	0.93	0.352	0.8307	1.6830	0.57	0.1512	−2.13	0.033	0.3361	0.9561
Aboriginal (ref. Non aboriginal)	0.72	0.1298	−1.81	0.070	0.508	1.0275	1.74	0.3921	2.48	0.013	1.1234	2.7105	1.24	0.3385	0.77	0.441	0.7218	2.1134
*Education level:*																		
Low education secondary (ref.)	0.62	0.0881	−3.35	0.001	0.472	0.8217	1.23	0.1984	1.27	0.203	0.8950	1.6857	0.98	0.2412	−0.09	0.926	0.6025	1.5852
Education level high	1.20	0.1188	1.8	0.072	0.984	1.4524	0.67	0.0771	−3.47	0.001	0.5364	0.8413	1.00	0.1925	0.01	0.993	0.6874	1.4597
*Material resources:*																		
Living in a house rented (ref. home owner)	0.63	0.0592	−4.96	0.000	0.520	0.7534	1.73	0.2262	4.21	0.000	1.3410	2.2374	2.14	0.3960	4.09	0.000	1.4845	3.0710
*Food insecurity:*																		
(ref. not food insecure) Moderately food insecure	0.69	0.1098	−2.31	0.021	0.509	0.9466	1.46	0.2891	1.89	0.058	0.9869	2.1491	1.65	0.4458	1.86	0.063	0.9737	2.8038
Highly Food insecure	0.66	0.1543	−1.78	0.076	0.418	1.0438	1.99	0.5477	2.51	0.012	1.1640	3.4162	2.28	0.7238	2.59	0.009	1.2233	4.2472
Has dental insurance (ref. no dental insurance)	3.67	0.3259	14.62	0.000	3.081	4.3647	0.27	0.0322	−10.94	0.000	0.2100	0.3375	0.09	0.0184	−12.03	0.000	0.0633	0.1371
Equivalized personal Income (log)	1.16	0.0652	2.73	0.006	1.0438	1.2999	0.86	0.0410	−3.27	0.001	0.7785	0.9393	0.84	0.0442	−3.26	0.001	0.7609	0.9343
Statistics:																		
k	28.00						28.00						28.00					
chi2	585.18						528.80						388.27					
p	0.00						0.00						0.00					
	*Observed*	*Bootstrap*			*Normal-based*		*Observed*	*Bootstrap*			*Normal-based*		*Observed*	*Bootstrap*			*Normal-based*	
	*Coef.*	*Std. Err.*	*z*	*P > |z|*	*[95% Conf. Interval]*		*Coef.*	*Std. Err.*	*z*	P > |z|	*[95% Conf. Interval]*		*Coef.*	*Std. Err.*	*z*	*P > |z|*	*[95% Conf. Interval]*	
CN	−0.003	0.0007	−4.00	0.000	−0.0044	−0.0015	0.024	0.0028	8.33	0.000	0.0181	0.0293	0.027	0.0041	6.46	0.000	0.0185	0.0346
CM	0.113	0.0050	22.70	0.000	0.1036	0.1232	−0.362	0.0215	−16.85	0.000	−0.4044	−0.3201	− 0.415	0.0412	−10.07	0.000	−0.4959	− 0.3343
HI	0.116	0.0050	23.37	0.000	0.1066	0.1261	−0.386	0.0213	− 18.16	0.000	−0.4276	−0.3443	− 0.442	0.0405	−10.91	0.000	−0.5209	− 0.3623

Bootstrap Normal-based standard errors. Data source: CCHS 2013–2014

^a^Estimates obtained after controlling for age, sex, marital status, urbanization level, occupational status

**Fig. 1:**
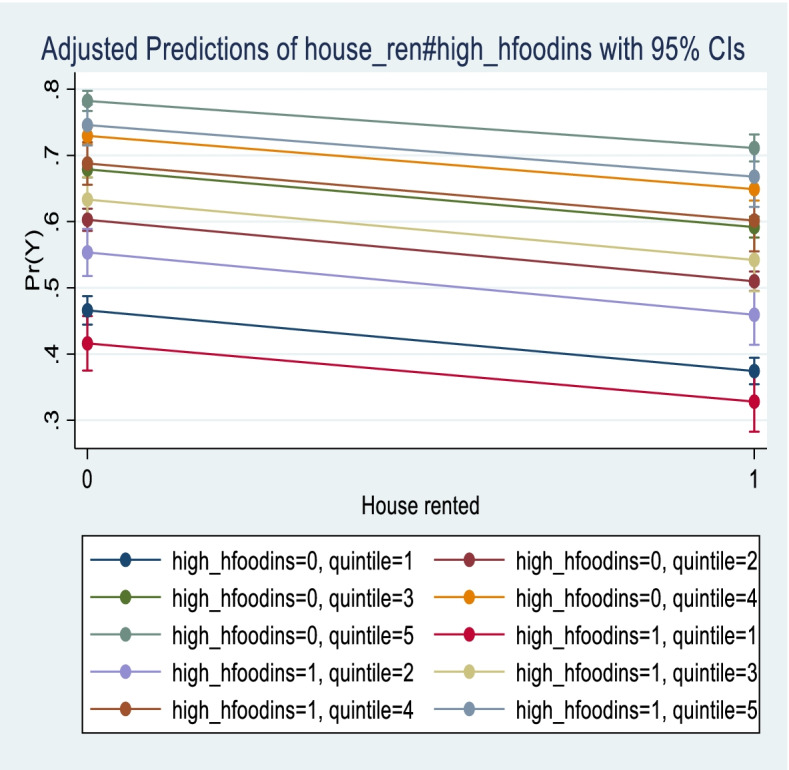
Average marginal effect of high food insecurity and house rented on the probability of seeing a dentist or an orthodontist over samples of different income quantiles (quintile). Legend: Data source: CCHS 2013–2014

**Fig. 2:**
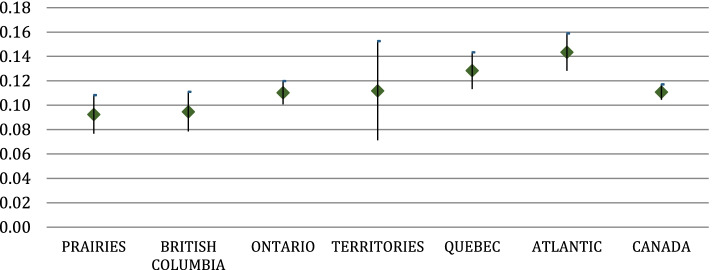
HI index estimation by Regions in Canada - Probability of having seen a dentist or an orthodontist in the last 12 months (estimates and their 95%confidence intervals). Legend: Data source: CCHS 2013–2014

The results show that food insecurity is an important non-need factor of both equity in the use of and access to dental care. In particular, food insecurity is associated with lower odds of using dental care (Table 2) and higher odds of reporting lack of access to dental care (Table 3). Importantly, another component of financial insecurity related to housing, as measured by having to pay rent and not owning a house, is an important non-need factor in both horizontal equity in the use (Table 2) and unmet access to dental care (Table 3). Overall, the probability of visiting a dentist would be higher if people were not living in high food insecurity and/or owned their dwelling (Fig. 1). There is pro-rich inequity in the probability of visiting a dentist or an orthodontist (Table 2). Inequities vary across jurisdictions. Atlantic (HI = 0.14; *P* = 0.008; CI:0.000 0.129) and Quebec (HI = 0.13; *P* = 0.000; CI:0.113 0.148) show the highest inequity in the probability of dental care, Ontario (HI = 0.11; *P* = 0.000; CI:0.103 0.123) and the Territories (HI = 0.12; *P* = 0.000; CI = 0.071 0.152) are similar to the national average (HI = 0.11; *P* = 0.000; CI:0.105 0.117), whereas British Columbia (HI = 0.09; *P* = 0.000; CI: 0.077 0.11) and the Prairies (HI = 0.10; *P* = 0.000; CI: 0.076 0.108) show values below the national average (Fig. 2, Table 2). In comparison with previous articles based on data for the period 2000–2005, dental care is still distributed in favour of the richest [5, 7, 9]. We obtained almost identical results by using either the more general question, “*Have you seen a dentist or orthodontist*”, available at the national level for all regions, or the more specific question, “*You reported that you have seen a dentist or orthodontist in the last 12 months: have you actually visited one?*” (Table 3). This confirms the robustness of results obtained at the national and regional levels without the extended set of variables on dental care. There are high inequities in unmet access to dental visits in Ontario (HI = -0.39, *P* = 0.000, CI:-0.4276 0.3443); again, high food insecurity and housing insecurity appear autonomous non-need factors contributing to the probability of reporting problems in access to dental care (Table 3). Our results show that the HI for unmet access does not change substantially using the richer set of variables from the special modules variables on dental care inclusion in Ontario if the reason for unmet visits is due only to costs (HI-0.44, *P* = 0.000, CI: − 0.5209 0.3623) (Table 3). Estimates for Ontario show that high and moderate food insecurity are associated with higher odds of reporting unmet access to dental care and lower odds of using dental care. Differently from the countrywide estimates, the odds of high food insecurity are higher than the odds of moderate food insecurity for both use of and unmet access to dental care. Similar to the nationwide estimates, living in a rented house is associated with lower odds of using dental care and higher odds of having problems in access (Table 3).

## Discussion

We can see from these results that not having food security and living in a rented house are associated with lower probability of using dental care, everything else being the same. These two variables also affect access to dental care in Ontario. Like in other countries such as Italy, we found that inequities in utilization are smaller than those in unmet access to dental care [37]. In the case of Ontario, inequities in access are three times higher than inequities in use of dental care. Estimates obtained by using the extended set of variables available for Ontario are similar to those obtained with the common set of variables available at the country level. This reinforces the results obtained with the core set of variables available nationwide. Our data are cross-sectional so we cannot infer causality. However, our evidence for Canada reinforces the view that reducing food insecurity in Europe and North America could counterbalance the negative effect of food insecurity on health care use [33, 46, 47]. Results are in line with previous evidence for Ontario showing that public spending on housing can offset the relationship between rising unemployment and food insecurity [47]. As a further test, we performed a decomposition analysis of the HI index for the probability of using dental care.^6^ This confirmed the results that housing and food insecurity are relevant determinants, together accounting for the largest contribution after income on the HI index (see Additional file 3).^7^

## Conclusions

Estimates of the HI (horizontal inequity) index for the probability of using dental care show that, overall, inequities in dental care use in Canada are persisting. This work adds to the literature by also estimating inequities in unmet access to dental care in selected areas of Canada for which data were made available (Ontario and NW Territories). As expected, in these areas inequities in access are much higher than inequities in use. Therefore, it seems important for Canada to systematically report on indicators not only on the use of dental care but also on unmet access to dental care. The methodology applied aimed at describing the contribution of other non-need factors to horizontal inequity than the traditionally considered such as income, education etc. We newly showed that both food insecurity and home ownership are non-need factors contributing to income-related inequity in dental care access and use. This study is based on cross-sectional data, and therefore inference on causality is limited. We cannot interpret this as a causal relationship from food and housing insecurity to dental care use, as these two variables can be influenced by unobservable characteristics that can also affect dental care use. We could not fully explore the links among insurance coverage, food insecurity, and home ownership due to data limitations at the national level. Future work will be targeted at analysing in detail such links by allowing the confounders and mediators of the relationship between each of the covariates and dental services to vary. Moreover, further work is required to see if and how extended insurance coverage for dental care could at least partially offset the difficulty of living in food insecurity and without home ownership in terms of improving access to dental care. Despite its limitations, this study favours the argument that it is important to tackle the food and housing insecurity of households in order to reduce existing inequities in access to and use of dental care, thus improving the overall equity performance of Canada’s health care system. There is a need for interventions aiming at improving equity in access to dental care, such as increasing public dental care coverage for fragile groups. Our study suggests that redistributing income may not be enough to reduce inequities in dental care use and access to dental care. What matters is to also act on other dimensions of purchasing power, such as housing tenure status and food security.

## Supplementary Information

**Additional file 1.** Sample definition. Description of the sample used for the analysis.

**Additional file 2.** Measuring horizontal inequity. Description of the methodology for measuring horizontal inequity.

**Additional file 3.** Decomposition of the HI Index for the probability of having visited a dentist during the last 12 months – CANADA (*). File containing a figure showing results from the HI decomposition.

## Data Availability

The data that support the findings of this study are available from Statistics Canada but restrictions apply to the availability of these data, which were used under license for the current study, and so are not publicly available. All responsibility for using the data stems on the Authors.

## Abbreviations

CCHS: Canadian Community Health Survey
FI: Food Insecurity
HI: House dwelling status
CI: Confidence interval
OR: Odds ratio
SE: Standard Errors
SES: Socio-economic status

## Footnotes

1 We also considered the number of consultations to a dentist/orthodontist. However, as indicated by previous evidence for Canada, results from the estimation of count models showed that SES determinants were not as relevant, and they are not reported here.

2 For mapping chronic conditions, we used a set of dummy variables, taking the value of 1 if individuals replied positively when asked if they were affected by a set of chronic conditions (under the premise that they should have been diagnosed by a health professional) and that they expected the conditions to last or had already lasted 6 months or more, and zero otherwise.

3 The number of items for the index is 14, the average interim correlation is 0.1637, and the Cronbach’s alpha is 0.73.

4 The questions are ascending in the severity of circumstances in terms of FI. Individuals were firstly asked “*Which of the following statements best describes the food eaten in your household in the past 12 months, that is, since [current month] of last year?*”. They were then asked whether they or other individuals in the family exhibited these behaviours: worried that food would run out before household members had money to buy more; found that the food bought just didn’t last, and there was no money to get more; couldn’t afford to eat balanced meals; relied on only a few kinds of low-cost food to feed a child because they were running out of money to buy food; couldn’t feed a child a balanced meal because they couldn’t afford it; were not eating enough because they just couldn’t afford enough food; ever cut the size of meals, ate less than they felt necessary, skipped meals, were hungry but didn’t eat, personally lost weight, or did not eat for a whole day; and finally, reflecting the worst situation, whether they had had to cut the size of any child’s meals, any child had ever skipped meals, any child was ever hungry but they just couldn’t afford more food, or any child ever did not eat for a whole day because there wasn’t enough money for food. In order to determine household food security status, responses to each question were coded as either “affirmative” or “negative”. Depending on the question, they had to reply either yes or no, or that those circumstances were often true, sometimes true, or never true in the past 12 months. Responses were then grouped into the three categories of food secure, moderately food insecure, and highly food insecure.

5 Observations for the Northwest Territories were insufficient for a disaggregated analysis in our sample. Therefore, we reported results for Ontario. In Ontario, 71% reported that they visited a dentist during the last 12 months, 29% reported that the last time they visited a dentist was more than 1 year ago, 11% between 1 year and 2 years ago, 5.3% from 2 to 3 years ago, 3.5% from 3 to 4 years ago, 1.7% from 4 to 5 years ago, 7.2% 5 years or more ago, and 1.14% had never visited a dentist.

6 Estimates for Ontario and other provinces showed similar results; therefore, they are not reported.

7 We are aware that more advanced techniques for decomposition are available and could be applied. However, our primary concern here was to see whether these determinants affected the use of dental care. Future analyses will compare results from more advanced techniques of decomposition.
