# Supplementary material for: Food insecurity, home ownership and income-related equity in dental care use and access: the case of Canada
Source: BMC Public Health. 2022 Mar 14;22:497. doi: 10.1186/s12889-022-12760-6 (PMC8919598; doi:10.1186/s12889-022-12760-6)
Supplement: Supplementary file 1 — Additional file 1. Sample definition. Description of the sample used for the analysis. [file 12889_2022_12760_MOESM1_ESM.docx]

Additional File **1**– Sample definition

**1-Initial survey sample** (n= 63,964):

**Criteria:**

Exclusions

Younger than 18 (- 4,982)

Missing variables (independent) (- 7,810)

**Remaining Sample**: 51,172

Exclusions:

Non missing observations for the dependent variable, i.e., i*ndividuals non-missing reply to*

*the question “Have you consulted with dentist or orthodontist in the last 12 months?”*  (-93)

**Final sample :** 51,079

**2- Dental modules:**

**Criteria:** Keep only observations common to dental module 1 and 2

(available only for the Province of **Ontario**)

From sample A above (n=51,172)

Exclusions

Keep only Dental Module 2 (DOOH2=1) observations (-28,484)

Remaining sample: 22,688

(Ontario 16,554, Manitoba 2,971, Saskatchewan 2,919, Nunavut 244)

Exclusions:

Respondents not from Ontario: (-5,235)

Remaining sample: 16,554

Data source: CCHS 2013-2014.
