# Supplementary material for: Food insecurity, home ownership and income-related equity in dental care use and access: the case of Canada
Source: BMC Public Health. 2022 Mar 14;22:497. doi: 10.1186/s12889-022-12760-6 (PMC8919598; doi:10.1186/s12889-022-12760-6)
Supplement: Supplementary file 2 — Additional file 2. Measuring horizontal inequity. Description of the methodology for measuring horizontal inequity. [file 12889_2022_12760_MOESM2_ESM.docx]

**Additional file 2: Measuring horizontal inequity**

A healthcare services distribution according to need can be analysed in terms of horizontal equity (Wagstaff and van Doorslaer, 2000). The idea is that people in the same state of ill-health have the same need and should be treated equally (ibid.). For the equity principle assumed here health care ought to be distributed according to need (Wagstaff and van Doorslaer, 2000).

For achieving the principle of horizontal equity, resources ought to be allocated according to needs, irrespective of personal characteristics not related with individuals’ needs, such as income or education. The needs of health care are, on average, related with the income distribution. Therefore, it will not be sufficient to measure inequality in medical care use but it will be necessary to quantify also the degree of inequality in needs (Wagstaff and van Doorslaer, 2000).

Using the Indirect standardization approach (Wagstaff and van Doorslaer, 2000) it is possible to generate the predicted value of medical care for each individual, that depends only on the needs of the population.

Horizontal equity in utilization can be assessed by comparing the actual distribution of health care use in relation to the expected distribution of healthcare use that would be obtained on the basis of need characteristics, irrespective of income or other non-need variables which are held constant and equal to the sample mean. It is worth noting that this does not require equality of utilisation. According to this principle, a distribution of dental care utilization is equitable if use and need distributions (by income) coincide. Horizontal equity in the use of health services is measured by the economic theory with indicators comparing the concentration of incomes with the distribution across income of the treatments analysed (O’Donnell et al., 2008)**.**

The estimation steps are the following (Masseria, 2010):

1) Measure if there is inequality in the actual distribution of use of dental care. To verify if people in equal need end up with similar use of dental care, irrespective of income.

2) Measure the level of inequality in the distribution of needs; because the needs of health care are, on average, related with the income distribution.

3) Measure the level of equity in access to dental care as a difference between (1) and (2).

We describe the steps hereinafter.

1. This study measures distributions of actual and needed use of dental care by income quintiles. These are

groups of equal size, each representing 20% of the total (adult) population, but ranked by their household

income from the poorest to the richest.

The distribution of dental care by income can be represented by the medical care concentration curve *L_M_(R).*

In the following figure, the horizontal axis is the cumulative proportion of the sample, *R*, ranked by income (from the worst-off to the most well-off), and the vertical axis is the cumulative proportion of dental care. The curve *L_M_(R)* plots the proportion of dental care used by each person (yi) ranked by income. If there is equality in dental care need, *LM(R)* coincides with the diagonal. If the delivery of dental care advantages the worse-off (better-off), *L_M_(R)* lies above (below) the diagonal.

*Horizontal inequity index*

The concentration index, *CM*, is a measure of the degree of inequality in the distribution of dental care that is associated with the income of individuals^[[1]](#footnote-1)^.

2) However, needs of health care are, on average, related with the income distribution. Using the indirect standardization approach, the predicted value of dental care for each individual that depends only on population needs; the predicted value indicates the amount of dental care that each individual would have received if she/he had been treated, on average, by the system, as others with the same need characteristics (Wagstaff and van Doorslaer, 2000). The “needed” health care use is computed by running a regression on all individuals in the sample, explaining medical care use (e.g. doctor visits or hospital nights) with a set of

explanatory variables. This means running a Logit regression equation for the probability of using dental care y which is equal to 1 if individual has used dental or orthodontal care; zero otherwise

y = 1 if y* > 0

y = 0 otherwise

[1] y* =α +β lninc +Σγ x +Σδ z +ε

Where:

*y* being the amount of dental care received by an individual *i* in a given period, our dependent variable. We distinguish between three types of explanatory variables: the (logarithm of) the household income of individual i (lninc), a set of need indicator variables (x) including demographic and morbidity variables (age, gender, having diabetes etc.), and then we have the other, non-need variables (z) (see table 1).

α, β, γ and δ are parameters and ε is an error term.

The concentration curve for needs, *L_N_(R)*, plots the need for dental care. There is equality in needs if *L_N_(R)* coincides with the diagonal. On the contrary, if the delivery of medical care advantages the worse-off (better-off), *L_N_(R)* lies above (below) the diagonal.

3) Estimating the level of horizontal inequity in the delivery of dental care is the last step. In this case, the predicted value of dental care for each individual that depends only on population needs is compared with the effective amount of dental care received by ranking each individual by income level. The equity principle of giving “equal treatment for equal need” will be violated if the share of dental care will not be equal to the share of needs. Thus, the degree of horizontal inequity can be measured by comparing the curves *L_M_(R)* and *L_N_(R).* The horizontal inequity index, *HI,* can be defined as twice the area between *L_N_(R)* and *L_M_(R)* and it is equal to the difference between *CM* and *CN*. The index *CM* and *CN* can alternatively be estimated by applying a “convenient” regression methodology (see Kakwani et al. (1997), and Wagstaff, van Doorslaer (2000a) and for a review Masseria, 2010).

When there is horizontal equity, *HI* will be equal to zero^[[2]](#footnote-2)^. Yet, there is horizontal inequity favouring the better off (worse-off) if the need concentration curve lies above (below) the dental care concentration curve. Whenever the *CM*, *CN* and *HI* are in favour of the better-off (worse-off) they have positive (negative) values.

This approach makes possible also to decompose the contribution of need and non-need variables as well as of the error component to the overall inequity in dental care (Wagstaff et al., 2003; O’Donnell et al, 2008). However, if the demand for health care is modelled using not linear estimation techniques, the decomposition method is not easily applicable (O’Donnell et al., 2008; Jones et al., 2007). A sensitivity analysis was performed comparing linear and non linear estimates of health care utilisation. As the linear results did not diverge from the non-linear results, the former coefficients were used for decomponsing inequity and calculating the contribution of each variable to total inequity. The contribution of each variable to total inequality is the sum of three factors: the relative weight of such variable (measured by its mean); its income distribution (indicated by the concentration index of the variable of interest); and the marginal effect on utilization of health care (linear regression coefficient, marginal effect); for example, if individuals with a high level of education are richer than the rest of the population (positive concentration index) and more likely to use dental care (positive marginal effect) their contribution to total inequality will be positive, whereas if they are less likely to use dental care (negative marginal effect), the contribution will be negative.

References

Jones A, Rice N, Bago d'Uva T, Balia S. Applied health economics. Abingdon: Routledge 2007.

Kakwani N, Wagstaff A, van Doorslaer E. Socioeconomic inequality in health: measurement, computation and statistical inference. Journal of Econometrics. 1997;77(1):87-104.

Masseria C. I determinanti delle disuguaglianze di salute: una rassegna di letteratura, in : L’equità nell’accesso alle cure sanitarie: prime stime e confronti interregionali, AUS-Regione Umbria, 2010.

O'Donnell O, van Doorslaer E, Wagstaff A, Lindelow M. Analyzing Health Equity Using Household Survey Data: A Guide to Techniques and their Implementation. Washington, D.C.: The World Bank; 2008.

Wagstaff A, van Doorslaer E. Equity in health care finance and delivery. In: Culyer AJ, Newhouse JP, editors. Handbook of Health Economics. Amsterdam: North-Holland; 2000. p. 1803-62.

Wagstaff A, van Doorslaer E, Watanabe N. On decomposing the causes of health sector inequalities with an application to malnutrition inequalities in Vietnam. Journal of Econometrics. 2003;112(1):207-23.

1. CM is based on L_M_(R), and it is defined as twice the area between L_M_(R) and the diagonal. [↑](#footnote-ref-1)
2. A zero index value is a sufficient but not necessary condition for implying no inequity. [↑](#footnote-ref-2)
