# Supplementary material for: Food insecurity, home ownership and income-related equity in dental care use and access: the case of Canada
Source: BMC Public Health. 2022 Mar 14;22:497. doi: 10.1186/s12889-022-12760-6 (PMC8919598; doi:10.1186/s12889-022-12760-6)
Supplement: Supplementary file 3 — Additional file 3. Decomposition of the HI Index for the probability of having visited a dentist during the last 12 months – CANADA (*). File containing a figure showing results from the HI decomposition. [file 12889_2022_12760_MOESM3_ESM.docx]

Additional File **3**- Decomposition of the HI Index for the probability of having

visited a dentist during the last 12 months – CANADA (*)

­

(*) Estimated % contributions obtained with linear decomposition.

Data source: CCHS 2013-2014.
